# Supplementary material for: Asymmetric introgression and thermal advantage jointly drive climate-mediated lineage turnover in a mixed-ploidy reed
Source: eLife. 2026 Sep 21;15:RP112160. doi: 10.7554/eLife.112160 (PMC13592815; doi:10.7554/eLife.112160)
Supplement: Supplementary file 2 [file elife-112160-supp1.docx]

 Supplementary File 1

**Table A** Heat tolerance metrics of different *Phragmites australis* genotypes, derived from chlorophyll fluorescence curve analysis.

|  | **Group** | ***T_crit_*** | ***T_50_*** | ***T_95_*** |
| --- | --- | --- | --- | --- |
| G2 | CN | 43.8 | 47.9 | 51.3 |
| NX16 | CN | 43.4 | 48.5 | 52.7 |
| TS5_7 | CN | 41.8 | 47.6 | 52.5 |
| XJ_BEJ | CN | 44.2 | 47.9 | 50.8 |
| YH04 | CN | 42.4 | 47 | 50.8 |
| ZY35 | CN | 41.8 | 47.1 | 51.5 |
| BH5_6 | FEAU | 45.6 | 49 | 51.8 |
| CD | FEAU | 44.4 | 48.3 | 51.2 |
| FZ2_10 | FEAU | 43.4 | 49.3 | 54.3 |
| LYG1_10 | FEAU | 43.2 | 47.3 | 50.8 |
| PJ5_4 | FEAU | 44.4 | 48.7 | 52.2 |
| YN01 | FEAU | 40.4 | 46.6 | 51.8 |

**Table B** Best-performing parameter combinations for the species distribution models of different *Phragmites australis* lineages.

|  | **FEAU** | **CN** | **SW** |
| --- | --- | --- | --- |
| fc | LQH | LQHP | LQ |
| rm | 0.5 | 0.5 | 1 |
| tune.args | fc.LQH_rm.0.5 | fc.LQHP_rm.0.5 | fc.LQ_rm.1 |
| auc.train | 0.945856 | 0.946841 | 0.964724 |
| cbi.train | 0.928 | 0.973 | 0.858 |
| auc.diff.avg | 0.057 | 0.082 | 0.078 |
| auc.diff.sd | 0.061 | 0.037 | 0.079 |
| auc.val.avg | 0.898 | 0.869 | 0.936 |
| auc.val.sd | 0.068 | 0.039 | 0.078 |
| cbi.val.avg | 0.757 | 0.731 | 0.517 |
| cbi.val.sd | 0.173 | 0.154 | 0.187 |
| or.10p.avg | 0.190 | 0.283 | 0.250 |
| or.10p.sd | 0.139 | 0.137 | 0.500 |
| or.mtp.avg | 0.056 | 0.046 | 0.250 |
| or.mtp.sd | 0.112 | 0.054 | 0.500 |
| AICc | 4446.910 | 4477.249 | 603.704 |
| delta.AICc | 0 | 0 | 0 |
| w.AIC | 0.776 | 1.000 | 0.658 |
| ncoef | 47 | 48 | 7 |

**Table C** Projected changes in suitable habitat distribution (%) for three *Phragmites australis* lineages in China under current and future climate scenarios (2061–2080).

| Lineage | Stage | High (%) | Middle (%) | Low (%) |
| --- | --- | --- | --- | --- |
| CN | Current | 9.4 | 28.5 | 62.1 |
| CN | 2061-2080 (SSP1-2.6) | 6.6 | 18.6 | 74.8 |
| CN | 2061-2080 (SSP5-8.5) | 16.5 | 20.0 | 63.4 |
| FEAU | Current | 6.6 | 24.5 | 69.0 |
| FEAU | 2061-2080 (SSP1-2.6) | 10.9 | 14.5 | 74.6 |
| FEAU | 2061-2080 (SSP5-8.5) | 25.2 | 18.1 | 56.7 |
| SW | Current | 3.3 | 6.6 | 90.1 |
| SW | 2061-2080 (SSP1-2.6) | 4.1 | 8.7 | 87.2 |
| SW | 2061-2080 (SSP5-8.5) | 5.3 | 10.7 | 83.9 |

**Table D** Collection details of *Phragmites australis* samples used for microsatellite analysis.

| **Province** | **Count** | **Longitude (°E)** | **Latitude (°N)** |
| --- | --- | --- | --- |
| Jilin | 14 | 125.8400 | 43.5880 |
| Liaoning | 8 | 121.876 | 41.000 |
| Xinjiang | 11 | 76.0060 | 39.4620 |
| Gansu | 48 | 100.2460 | 39.1370 |
| Ningxia | 26 | 105.8120 | 38.0220 |
| Qinghai | 97 | 97.6860 | 37.1930 |
| Shandong | 68 | 118.8040 | 36.8310 |
| Shanxi | 1 | 110.9970 | 35.0110 |
| Henan | 1 | 114.2960 | 34.8010 |
| Hebei | 39 | 114.0640 | 32.2040 |
| Jiangsu | 23 | 119.4520 | 32.1750 |
| Shanghai | 11 | 121.7850 | 31.5810 |
| Hubei | 5 | 111.4710 | 30.9560 |
| Sichuan | 9 | 104.0870 | 30.6200 |
| Chongqing | 15 | 106.4920 | 29.5000 |
| Hunan | 21 | 113.0650 | 29.4070 |
| Zhejiang | 17 | 120.9990 | 29.1550 |
| Jiangxi | 49 | 115.7290 | 28.0650 |
| Yunan | 10 | 102.6600 | 25.0130 |
| Fujian | 10 | 118.5040 | 24.9840 |
| Guangdong | 7 | 113.5500 | 22.6440 |
| Guangxi | 5 | 109.1340 | 21.5760 |

**Table E** Characteristics of the microsatellite markers used in this study, including marker name, chromosomal location, quality metrics, sequence, and primer information.

| **Marks** | **Chromosome** | **state** | **motif** | **motif_length** | **target_seq_length** | **primer_F** | **primer_R** |
| --- | --- | --- | --- | --- | --- | --- | --- |
| SSR_3_ | PaChr3 | Good | CA | 2 | 197 | ATTGAATCCACACGTTTCCG | CCATGTGTTAATGTTGTCC |
| SSR_4_ | PaChr18 | Good | CA | 2 | 146 | CAACTCCGTGAATGACATGC | CAGTTTGTGCACTAATGGAC |
| SSR_5_ | PaChr10 | More than 4 alleles (>10 samples) | CA | 2 | 166 | CTTCCTAGGTCAGTATCATCC | GTGGCAGCTGATTGATTTGG |
| SSR_6_ | PaChr12 | Good | TG | 2 | 208 | CTCATGCATCACTTCACAGG | ACACGGACCTAACATCAACC |
| SSR_7_ | PaChr5 | More than 4 alleles (>10 samples) | CA | 2 | 181 | CAAGCATTCTAGTAGTAGC | GTTGCAGCAAGTATTTGG |
| SSR_AU_1128_ | PaChr12 | Good | CGG | 3 | 119 | AGACTCGCCTGCCAGATTTC | CACACTTCTTCTCCCGGCTT |
| SSR_AU_1284_ | PaChr1 | Good | CGC | 3 | 130 | TGCAACCGACGTATGCTTCT | GCCGCGCTTGAAGATAAGGT |
| SSR_AU_151_ | PaChr11 | Good | AGC | 3 | 152 | CGAAGCGTCCCACGAGAG | TTGCTTCTGACGCTACTGGG |
| SSR_AU_1531_ | PaChr22, PaChr10 | More than 4 alleles (>10 samples) | ACA | 3 | 113 | GGAGTGCAAGATGACTGTGC | CCCTGGATCTTGGCCTTCTT |
| SSR_AU_1825_ | PaChr13 | Good | CTG | 3 | 118 | TCAGCCTACTGTTGAAGATGGT | TTCAGTTTCGACTGCCTGCT |
| SSR_AU_1891_ | PaChr4 | Good | AGG | 3 | 103 | GCGTCGATAAGGATGGGGAG | GTCACCCTCTTCAGCCACTC |
| SSR_AU_2008_ | PaChr3 | Good | GCT | 3 | 122 | CGAAACCAAGCGAGCATCAG | AGATCTGCGTCTTCGTCCAC |
| SSR_AU_2013_ | PaChr3 | More than 4 alleles (>10 samples) | GGT | 3 | 108 | GATGGCGGTAGAGGTGACAG | GCCAGAGTATCGGTCACCAC |
| SSR_AU_2160_ | PaChr14 | Good | GTG | 3 | 143 | TGCTTCTGCTCCTCCTGTTC | CCAGTACCAGTACCACCACG |
| SSR_AU_2296_ | PaChr6 | Good | TTC | 3 | 159 | GGAACAGGGCTAATTTCCAAGC | ATAGCCGCAGCCATTAGGAC |
| SSR_AU_2812_ | PaChr13 | Good | AGG | 3 | 115 | CCAGCAAGTGAAGGATCCGA | CTCTCCCCCTTGCACCAAAT |
| SSR_AU_293_ | PaChr16 | Good | CCT | 3 | 157 | AAGCAGCACCCAGATCACTC | TGCTCGCTCCAACAAGACAA |
| SSR_AU_3292_ | PaChr12 | Good | GAG | 3 | 144 | GACCTCTCCGCGTTCTACG | CTGGGGGATCTGCAGGAAG |
| SSR_AU_4223_ | PaChr16 | Good | TTC | 3 | 145 | CGGCCGTAATCCAATCAATGT | AGGAGGAGAAAAAGGGTGCG |
| SSR_AU_4297_ | PaChr3 | Good | AGG | 3 | 198 | ATCGCTTCCTATTGGCCTCG | CTGCACGTCCTCCATCTCC |
| SSR_AU_448_ | PaChr25 | Good | GAA | 3 | 120 | GCCCAAGGTCAGGTTTGGTT | GCCCTTCTCTTCCTCCCTCT |
| SSR_AU_4737_ | PaChr11 | Good | CTC | 3 | 121 | TACGAGCCGACTCCCAGTAA | CTCCTCCTCTCTCTCGGCTT |
| SSR_AU_643_ | PaChr3 | Good | GTC | 3 | 126 | GCCCTCTGCTTAGCTACCAT | TCTCACGCAGGAGGGGAG |
| SSR_AU_656_ | PaChr5 | Failed amplifications (>10 samples) | CAG | 3 | 172 | AGATCACCTCCGATCCTCCC | GTGTCCCCGAATGGTGAGG |
| SSR_CO_15_ | PaChr12 | More than 4 alleles (>10 samples) | CCT | 3 | 113 | CTCCACAAGGTCCTCCACAC | GCCCGTACGGTGATCCAATA |
| SSR_CO_18_ | PaChr15 | Good | TCC | 3 | 126 | GTAGCCTCCCTTGCGGTAG | GAGGAGGAACCGAGGAGGAT |
| SSR_CO_19_ | PaChr19 | More than 4 alleles (>10 samples) | AG | 2 | 116 | ATGTGCGCTTCGAGAATGGA | GGGTTCACGGTGCGTCTG |
| SSR_CO_23_ | PaChr3 | Good | TCT | 3 | 118 | CCTCGACAGGAACCTCCCTA | TAAGAAACCTGCAGGGAGGC |
| SSR_CO_25_ | PaChr8, PaChr14(2), PaChr7(3) | More than 4 alleles (>10 samples) | AG | 2 | 124 | CGGAGGGGTTTATTCCAGGC | AGGGGTGGAGAGGAAGTATCA |
| SSR_CO_27_ | PaChr18 | More than 4 alleles (>10 samples) | TC | 2 | 144 | TTCCCATGTGCTTGTCTCGC | AGATTGAGCAGCCTGTGACG |
| SSR_CO_32_ | PaChr12 | Good | CTAC | 4 | 124 | TGTGTATGCCTGTCACCCAA | ACAAAATCAGCACATCAGCACA |
| SSR_CO_33_ | PaChr6 | Good | TC | 2 | 152 | TCTTCCTCCTCTGGTTCGGT | TGGTACGGTGTTTCGTGGAG |
| SSR_CO_38_ | PaChr6 | Good | GA | 2 | 160 | CAAGCCAAACAAAGCCACCA | TGTGAGGCTCCATGTGCTTT |
| SSR_CO_42_ | PaChr11 | Good | AAGA | 4 | 128 | GGACCTCTTGTCTGAAACCCA | TCGCAAAACAGAAAAGGAGGC |
| SSR_CO_50_ | PaChr23 | Good | AGG | 3 | 108 | GTGCTGGGGGAAGGAGTTC | CTCTCCTCCCTCCACCTCC |
| SSR_CO_69_ | PaChr12 | Good | CTC | 3 | 105 | CCTCCTCCTCTCCCATCTCC | AGAAGGCTAAGGGCAAGGAC |
| SSR_CO_7_ | PaChr20 | Good | TG | 2 | 124 | TGTAGTAATTTGTCGGTTTGCCA | CAACCGAGCAGTAGCCTTGA |
| SSR_CO_8_ | PaChr4 | Failed amplifications (>10 samples) | TGC | 3 | 138 | CAAGTGGTGGTGGTGCCTC | CATTCCGCGATTCTGCCAC |
| SSR_CO_9_ | PaChr3 | Good | CCA | 3 | 163 | CGAGGCCTCCGACTACATC | ACAACGAATGCACGAATCGC |
| SSR_EU_1202_ | PaChr20 | Good | GCG | 3 | 101 | CTTCTCTGCATCGCTTTGCC | ATCTCCACTGCCCTCATCCT |
| SSR_EU_1624_ | PaChr1 | Good | CTC | 3 | 179 | GCTTCGTCTCGCCTAAACCT | CTTCTCGAAGGTGGACTCCG |
| SSR_EU_1667_ | PaChr7 | Good | TCC | 3 | 158 | GCGGTCTAGCTGTTGGATGT | CACAGCGACCAAGCAAAGAG |
| SSR_EU_2523_ | PaChr6 | More than 4 alleles (>10 samples) | GTG | 3 | 129 | ACAGTGGCCGAGAACATCAG | CTGCAACACAACACGGTGAG |
| SSR_EU_2685_ | PaChr14 | More than 4 alleles (>10 samples) | TTC | 3 | 152 | CTCAGCCGCGTCTTCTTCTT | CAACAACACCACACCGACAA |
| SSR_EU_2709_ | PaChr12, PaChr14 | Good | GCA | 3 | 168 | GTGCAGGTGGCCGATGAT | AATGCGACCACACCTCTAGC |
| SSR_EU_3127_ | PaChr9, PaChr12 | More than 4 alleles (>10 samples) | GAG | 3 | 146 | ATGGCAATGGAACGGAGAGG | AACATCCCAAGCGTAGGTGG |
| SSR_EU_3391_ | PaChr23 | Good | GCG | 3 | 128 | CCGCCACCAACTCTTTCTCT | GTAGCTCTTGGGGTGCGAG |
| SSR_EU_3553_ | PaChr2 | Failed amplifications (>10 samples) | ACC | 3 | 150 | GAGTGCAACCCCAACTCCA | GGAGATGTTGCCGAAGACCT |
| SSR_EU_3761_ | PaChr19, PaChr1, PaChr2(2), PaChr6, PaChr18 | Failed amplifications (>10 samples) | CTC | 3 | 175 | AGGAAGCGGAACCCCCTG | CGAAGGACAAGGAGGAGCAG |
| SSR_EU_3780_ | PaChr15 | Good | CTG | 3 | 113 | TAGCAGCTGCAAACCCATGA | ACGAGTTGTTGCTGAGGAGG |
| SSR_EU_4001_ | PaChr4 | Good | GCT | 3 | 109 | AGCTTTCAGACTGTCATGGTCA | GCAGCACAGAAAGGTCATGC |
| SSR_EU_4063_ | PaChr18 | Good | CGT | 3 | 176 | TAGGGGCAGTCGTCGATCTT | AGCTTTACCTTCGTGGCTCC |
| SSR_EU_4112_ | PaChr5 | Good | GAC | 3 | 216 | ACCGTGAAACACCCCATCTC | GGAAAGGTCGTACTCGTCGG |
| SSR_EU_4515_ | PaChr3 | Good | GGA | 3 | 101 | ATCTGTCGGTCGTTCGTTCG | GTATTGCGTGGCCTAGGGTT |
| SSR_EU_643_ | PaChr8 | Good | ATG | 3 | 190 | TGTGGCATTCCAGTAGCAGT | CCATCATCGTCTTCTTTGGCAC |
| SSR_EU_811_ | PaChr5 | Good | GCG | 3 | 179 | AGGAGGGATCCGGAGTTCAA | GGAGTTGAGCTTGACGACGA |
| SSR_EU_914_ | PaChr6 | Failed amplifications (>10 samples) | GCT | 3 | 142 | CCACCTCACCTCTTGGCAAT | CACGAGAGGGTGGTGTGTTT |
| SSR_EU_997_ | PaChr2 | Good | TTG | 3 | 114 | CATCCCTAGTCTATCCCAACTTGT | GCAACAGGACCGGTCACATA |

**Table F** *Phragmites australis* samples used in the common garden experiment, detailing their designated name, haplotype, and geographical origin.

| **ID** | **Garden** | **Group** | **Haplotype** | **Latitude** (°N) | **Longitude** (°E) |
| --- | --- | --- | --- | --- | --- |
| YRD9 | Jinan-Panjin | CN | O | 38.0037 | 118.9496 |
| YRD8 | Jinan-Panjin | CN | O | 37.9719 | 118.8778 |
| YRD7 | Jinan-Panjin | CN | O | 37.8447 | 118.9930 |
| YRD5 | Jinan-Panjin | CN | O | 37.8338 | 119.0382 |
| YRD4 | Jinan-Panjin | CN | O | 37.7950 | 118.8973 |
| YRD3 | Jinan-Panjin | CN | O | 37.7458 | 119.1342 |
| YRD2 | Jinan-Panjin | CN | O | 37.7458 | 119.1342 |
| Salt1 | Jinan-Panjin | CN | O | 38.0458 | 118.6777 |
| DW1 | Jinan-Panjin | FEAU | P | 37.7929 | 118.4386 |
| CN2028 | Jinan-Panjin | CN | O | 41.2069 | 122.0279 |
| CN2026 | Jinan-Panjin | FEAU | P | 41.2069 | 122.0279 |
| CN2024 | Jinan-Panjin | FEAU | P | 41.0892 | 122.0578 |
| CN2023 | Jinan-Panjin | FEAU | P | 36.4551 | 120.6805 |
| CN2021 | Jinan-Panjin | FEAU | P | 37.0676 | 118.0605 |
| CN2020 | Jinan-Panjin | FEAU | P | 37.0712 | 118.0608 |
| CN2019 | Jinan-Panjin | FEAU | P | 37.0712 | 118.0607 |
| CN2018 | Jinan-Panjin | CN | O | 37.0484 | 118.0653 |
| CN2017 | Jinan-Panjin | CN | O | 37.0484 | 118.0653 |
| CN2016 | Jinan-Panjin | FEAU | P | 30.2593 | 120.0623 |
| CN2014 | Jinan-Panjin | FEAU | P | 30.1652 | 120.0942 |
| DY3_10 | Qingdao-Shanghai | FEAU | P | 37.8306 | 119.0783 |
| LANZHOU | Qingdao-Shanghai | CN | O | 36.2049 | 103.7024 |
| PJ1_4 | Qingdao-Shanghai | FEAU | P | 40.8977 | 121.7893 |
| QH05 | Qingdao-Shanghai | CN | M | 35.8456 | 102.8543 |
| SH1_8 | Qingdao-Shanghai | FEAU | P | 31.6844 | 121.6522 |
| TS1_3 | Qingdao-Shanghai | CN | O | 39.0254 | 118.3356 |
| TS2_1 | Qingdao-Shanghai | CN | O | 39.0414 | 118.2834 |
| TS3_1 | Qingdao-Shanghai | CN | O | 39.0613 | 118.2254 |
| TS4_3 | Qingdao-Shanghai | CN | O | 39.0774 | 118.2062 |
| TS5_7 | Qingdao-Shanghai | CN | O | 39.0987 | 118.1895 |
| WZ1_5 | Qingdao-Shanghai | FEAU | P | 28.0054 | 120.7837 |
| WZ4_7 | Qingdao-Shanghai | FEAU | P | 27.9836 | 120.7745 |
| WZ5_10 | Qingdao-Shanghai | FEAU | P | 27.9852 | 120.7671 |
| XJ_BEJ | Qingdao-Shanghai | CN | M | 39.4616 | 76.0062 |
| YC4_4 | Qingdao-Shanghai | FEAU | P | 33.6182 | 120.4933 |
| YC5_10 | Qingdao-Shanghai | FEAU | P | 33.6242 | 120.4817 |
| YUNCHENG | Qingdao-Shanghai | CN | O | 35.0106 | 110.9972 |
