# Supplementary material for: Asymmetric introgression and thermal advantage jointly drive climate-mediated lineage turnover in a mixed-ploidy reed
Source: eLife. 2026 Sep 21;15:RP112160. doi: 10.7554/eLife.112160 (PMC13592815; doi:10.7554/eLife.112160)
Supplement: Peer review history

# Editor report: eLife Assessment

**DOI:** 10.7554/eLife.112160.3.sa0

**Authors:** Alexandre Fournier-Level

**Affiliations:** The University of Melbourne Australia

**Keywords:** Convincing, Solid, Important

The report by Liu and colleagues reports on an analysis of environmental adaptation across diverse lineages of the grass *Phragmites australis* differing by their level of ploidy. These results represent **important** findings for understanding the environmental adaptation of species complexes with mixed ploidy and the analysis reports **solid** evidence that lineages with distinct levels of ploidy occupy different climate niches. The use of regional survey in tandem with common garden experiment represents a **convincing** approach to suggest a correlation between ploidy and climate adaptation. This manuscript will be of interest to a broad community of ecological genomicists interested in how structural variation in gene dosage potentially affects the pattern of adaptation.

# Referee report: Reviewer #1 (Public review)

**DOI:** 10.7554/eLife.112160.3.sa1

Summary:

The article is testing the relative advantages of plant lineages with differing ploidy and admixture across environmental gradients. The results show that intraspecific variation in ploidy and admixture between lineages impacts plant traits that may enable persistence and range expansion.

Strengths:

Suitable marker panel size and strong results that include attempts to analyse mixed ploidy level data which is a challenge.

Weaknesses:

The sample sizes of the common garden experiments are very low making it difficult to draw robust conclusions.

# Author comment: Author response

**DOI:** 10.7554/eLife.112160.3.sa2

**Authors:** Lele Liu, Wenyi Sheng, Yuhui Wang, Lele Lin, Cui Wang, Huijiaq Song, Yaolin Guo, Weihua Guo

**Affiliations:** Shandong University Qingdao China; Shandong University Qingdao China; Shandong University Qingdao China; Chinese Academy of Forestry Beijing China; Shandong University Qingdao China; National Natural History Museum of China Beijing China; Louisiana State University Agricultural Center Baton Rouge United States; Shandong University Qingdao China

The following is the authors’ response to the original reviews.

Overview of Revisions

We thank the editors and reviewers for their constructive and insightful comments, which have substantially improved the manuscript. We have carefully addressed every point raised. The major revisions include:

(1) Methods 2.1: completely reorganized to clarify the allotetraploid genome structure of *Phragmites australis*, the rationale for single-chromosome-anchored microsatellite markers, the maximum distinguishable alleles per ploidy level, and the conservative Ploidies(mydata) <- 4 setting in polysat.

(2) Methods 2.3 / Results 3.3 / Discussion 4.4: clarified common garden sample sizes, added Cohen's *d* effect sizes, and acknowledged the correlational nature of the lineage-level comparisons.

(3) Introduction: added a new paragraph on the eco-evolutionary significance of gene flow in mixed-ploidy systems, and another paragraph emphasizing the novelty of integrating SDMs with physiological and common garden experiments.

(4) Discussion 4.1 and 4.4: reframed all "polyploidy-driven" language to "polyploidy-associated", explicitly acknowledging that ploidy is confounded with genetic background and was not experimentally manipulated.

**Public Reviews:**

**Reviewer #1 (Public review):**

(R1-P1) Inadequate explanation of allele dosage for ploidy levels

Inadequate explanation of allele dosage for ploidy levels, some of which do not match the allele counts expected for genome copy number.

We appreciate this comment and have substantially revised Methods 2.1. The key clarifications are:

(A) Marker specificity: All 42 microsatellite markers were aligned to the *P. australis* reference genome, and each marker mapped to a single unique chromosome (Table S2). This confirms that each marker amplifies a locus specific to one subgenome only. Therefore, in tetraploids each marker detects at most two alleles (the two homologous copies of that chromosome from one subgenome), while in octoploids (autopolyploid derivatives with four copies of the same chromosome) each marker detects up to four alleles.

(B) Conservative ploidy setting: We set Ploidies(mydata) <- 4 for all samples in polysat because the exact ploidy of many samples could not be confidently assigned a *priori*. This uniform treatment is conservative: for actual tetraploids, the two unobserved "copies" are scored as null; for actual octoploids, all four detected alleles are accommodated.

(C) Dosage estimation: Allele dosage was estimated from high-coverage sequencing read counts (mean >5,000× per locus per sample) using the SSRSeq V1.1 pipeline (Cui et al., 2022), which applies stutter correction, amplification bias correction, and ploidy-optimized dosage calling, not inferred from allele presence/absence alone.

Methods 2.1, second paragraph onward

“*Phragmites australis* has a base allotetraploid genome. All 42 microsatellite markers used in this study were aligned to the *P. australis* reference genome, and each marker mapped to a single unique chromosome (Table S2), confirming that each marker amplifies from one subgenome only. Therefore, in tetraploids each marker detects at most two alleles (the two homologous copies of that chromosome from the target subgenome) while the homologous region from the other subgenome is not amplified (Saltonstall, 2003). In Asia, the prevalent octoploids are most likely autopolyploid derivatives of tetraploids, carrying four homologous copies of the same chromosome and thus capable of up to four distinguishable alleles per locus (Liu et al., 2022; Wang et al., 2024). Hexaploid individuals are rare and occur primarily in contact zones, likely originating from inter‑lineage hybridization (Wang et al., 2024).

In practice, the 42 selected markers very rarely produced more than four alleles in any single individual (Table S2), consistent with a ploidy ceiling of octoploid. Because the exact ploidy of many samples could not be confidently assigned a *priori* (ploidy was inferred from a combination of chloroplast haplotype, geographic origin, and flow cytometry from prior studies; Lambertini et al., 2020; Liu et al., 2022), we consistently set Ploidies(mydata) <- 4 in the polysat R package (Clark & Jasieniuk, 2011), treating every individual as having four homologous copies. This uniform treatment is conservative: for an actual tetraploid (two copies per locus), the two unobserved "copies" are simply scored as null (missing data) in the dosage matrix; for an actual octoploid, all four detected alleles are accommodated. The allele dosage itself was estimated from high-coverage sequencing read counts (mean >5,000× per locus per sample) using the SSRSeq V1.1 pipeline (Cui et al., 2022), not inferred from allele counts alone.”

(R1-P2) Common garden setup and sample sizes unclear

The setup and sample sizes of the common garden experiments are very unclear. The numbers implied are extremely low to draw robust conclusions.

We agree that the original description was insufficiently detailed. We have made three modifications:

(A) Methods 2.3: clarified that each population contributed one rhizome segment planted in one pot (one biological replicate per population per site), and emphasized that the key inference rests on the direction and consistency of differences across four climatically distinct sites rather than on significance at any single site.

(B) Results 3.3: added Cohen's *d* effect sizes (verified from the raw data), showing that where lineage differences are present, they are biologically substantial (*d* = 1.10–1.43 at three of four sites).

(C) Discussion 4.4: added a paragraph acknowledging the limited number of populations per lineage and the need for future confirmation with a larger panel.

Methods 2.3

“(1) A previously published common garden experiment (Song et al., 2021) conducted in 2017 across Jinan (36.43°N, 117.45°E) and Panjin (41.20°N, 122.02°E), using CN (n = 11) and FEAU (n = 9) lineages, for which we determined the haplotype information of all samples; (2) A new common garden experiment established in 2021 across Qingdao (36.36°N, 120.69°E) and Shanghai (30.20°N, 121.29°E), with CN (n = 9) and FEAU (n = 8) lineages (Table S3). Each rhizome segment (2–3 buds per segment, one segment per population) was transplanted into an individual 20 L pot, yielding one biological replicate per population per site. Although the number of populations per lineage is modest, the key inference rests on the direction and consistency of lineage differences across four climatically distinct sites rather than on the statistical significance at any single site.”

Results 3.3

“Similarly, plant height was significantly greater in the FEAU lineage than in the CN lineage in Jinan, but not in the other common gardens (Figure 3C). Effect sizes for total biomass were large in Jinan (Cohen's *d* = 1.10), Panjin (*d* = 1.12), and Qingdao (*d* = 1.43), but negligible in Shanghai (*d* = 0.37), confirming that the lineage differences, where present, are biologically substantial.”

Discussion 4.4

“We also acknowledge that the common garden experiments, while replicated across four climatically distinct sites, involved a limited number of populations per lineage (9–11 CN and 8–9 FEAU), which constrains our ability to fully separate lineage-level effects from population-level variation. The consistent direction of biomass differences across three of four sites, supported by large effect sizes, nonetheless provides robust evidence for a lineage-level performance advantage that merits further confirmation with a larger, more geographically representative panel of populations.”

(R1-P3) How allele dosage is determined

Unclear how allele dosage is determined. Given it's so central to many analyses, it would be useful to see how this is done rather than use a citation.

We agree that a self-contained description is warranted. We have rewritten the relevant paragraph in Methods 2.1 to describe the three core steps of the SSRSeq V1.1 pipeline (Cui et al., 2022): (i) stutter correction based on empirically estimated slip ratios; (ii) amplification bias correction across alleles of different repeat lengths; and (iii) ploidy-adjusted allele dosage calling. The pipeline's source code and full documentation are available at https://github.com/ccoo22/SSRseq_count.

Methods 2.1

“Microsatellite genotyping was performed using the SSRSeq V1.1 pipeline (Cui et al., 2022; https://github.com/ccoo22/SSRseq_count). Briefly, the pipeline takes the per-locus per-sample read count table generated from high-throughput sequencing and processes it through three core steps fully described in Cui et al. (2022): (i) stutter correction, which reallocates a fraction of reads from each allele to its adjacent repeat class based on empirically estimated slip ratios; (ii) amplification bias correction, which normalizes read counts across alleles of different repeat lengths using locus-specific bias coefficients; and (iii) allele dosage calling, which selects the maximum number of alleles consistent with the specified ploidy (four in this study) and assigns integer dosages (0–4) by comparing corrected read ratios to a ploidy-adjusted threshold optimized to minimize both allelic dropout and false positives. The final output is a genotype matrix with integer allele dosages for all samples and loci, which was used directly as input to the polysat R package for subsequent population genetic analyses.”

**Reviewer #2 (Public review):**

(R2-P1) Polyploidy has no causal evidence; confounded with genetic background

First, no data support the claims that polyploidy has any causal effect. The ploidy levels are, in fact, completely confounded with other genetic differences, so it is not possible to eliminate genetic variation, independent of ploidy, as the causative factor. As the authors note, ploidy was not manipulated in the reported experiments. Thus, the focus on polyploidy in the introduction and elsewhere distracts from the novel and informative experiments that were conducted.

We fully acknowledge this critical limitation and thank the reviewer for this important critique. We have revised the manuscript at four locations to reframe all claims from "polyploidy-driven" to "polyploidy-associated" and to explicitly state that ploidy is confounded with lineage identity and was not experimentally manipulated.

Abstract (last sentence)

“These results demonstrate that climate change interacts with intraspecific variation among polyploidy-associated lineages, manifested through differences in thermal tolerance, biomass production, and asymmetric gene flow, to drive potential lineage replacement within a native range”

Introduction (polyploidy paragraph)

“The octoploid FEAU lineage is distinguished from its tetraploid relatives not only by ploidy level but also by its distinct evolutionary history, genomic background, and geographic origin. Polyploidy has been shown in other systems to generate genetic novelty, alter gene expression, and enhance physiological stress tolerance (Bureš et al., 2024; Cheng et al., 2021; Kolář et al., 2017; Van de Peer et al., 2017), potentially pre-equipping polyploid lineages to occupy new geographical ranges and endure environmental shifts (Cheng et al., 2021; López-Jurado et al., 2019). The FEAU lineage's superior thermal tolerance and biomass are consistent with such polyploidy-associated effects, although ploidy is correlated with, rather than experimentally separable from, the broader genetic identity of each lineage.”

Discussion 4.1 (title and opening paragraph)

“Our findings demonstrate that the octoploid FEAU lineage of *P. australis* possesses greater heat tolerance and biomass production than the tetraploid CN lineage. Under a high emission scenario (SSP5-8.5), the projected suitable habitat for the FEAU lineage expands by 18.6%, while the CN lineage exhibits a much smaller relative increase. Several non-mutually-exclusive mechanisms could explain these lineage-level differences, including increased gene dosage from whole-genome duplication, divergent selection histories, and/or standing genetic variation in thermal tolerance loci unlinked to ploidy (Bures et al., 2024; Cheng et al., 2021; Van de Peer et al., 2017). Our data cannot fully partition these factors, but the strong association between lineage identity and both physiological performance and projected range dynamics highlights the importance of incorporating intraspecific lineage information into ecological forecasts, regardless of the ultimate causal mechanism.”

Discussion 4.4 (limitations paragraph)

“Crucially, ploidy was not experimentally manipulated in this study; it is inherently confounded with the distinct evolutionary history and genomic background of each lineage. While the observed thermal tolerance and biomass differences are consistently associated with the octoploid FEAU lineage, we cannot formally exclude the possibility that these traits are driven by genetic factors independent of ploidy *per se*. Future studies using experimental approaches that can partition ploidy effects from lineage-specific genetic effects, such as common gardens with synthetic polyploids or transcriptomic analyses comparing gene expression dosage responses, are needed to strengthen causal inference (Wei et al., 2020). Similarly, the common garden results should be interpreted as lineage-associated rather than ploidy-causal performance differences. The potential role of admixture in facilitating the adaptive introgression of heat tolerance alleles also warrants deeper investigation (Suarez-Gonzalez et al., 2018).”

(R2-P2) SDMs treat lineages as homogeneous entities

Second, the manuscript indicates that intraspecific variation is critical for the evolutionary potential of a species to respond to environmental change, but intraspecific variation is seldom considered in species distribution models... the manuscript performs species distribution modeling on a small number of sub-specific lineages, essentially treating them as homogeneous "species"—thus the analysis commits the same oversimplification that the manuscript highlights, but does so at a finer evolutionary scale than species.

We acknowledge this important limitation and agree that it deserves explicit discussion. While disaggregating the species into three major genetic lineages is a step forward from species-as-monolith approaches, within-lineage variation in thermal tolerance, growth, and dispersal capacity is plausible given the broad geographic ranges of the CN and FEAU lineages. We have added a new paragraph in Discussion 4.4 to address this point.

Discussion 4.4 (new paragraph)

“We also recognize that our SDM approach, while disaggregating the species into three major genetic lineages, still treats each lineage as a homogeneous entity. This simplification parallels—albeit at a finer scale—the species-as-monolith assumption that we critique in the Introduction. Within-lineage variation in thermal tolerance, growth, and dispersal capacity is plausible, particularly given the broad geographic ranges of the CN and FEAU lineages. By modelling each lineage as a uniform group, our projections may overestimate the precision of range forecasts and underestimate the evolutionary potential of standing variation within lineages (Chardon et al., 2020). Future frameworks that incorporate trait variation at multiple hierarchical levels (population, lineage, ploidy) will be necessary to capture both the adaptive potential and the ecological constraints that shape species' responses to climate change.”

(R2-P3) Asymmetric introgression and thermal tolerance lack context in Introduction and Discussion

The title suggests that asymmetric introgression and thermal tolerance are the most important findings of the work. However, the introduction contains no explanation of the potential importance of gene flow (other than to say that asymmetric gene flow was suggested by some preliminary analyses), and the discussion offers only a limited explanation of either the potential mechanisms underlying the asymmetric gene flow or its importance for the long-term evolution of the species.

We agree that the evolutionary significance of asymmetric gene flow was underdeveloped. We have added two substantial new passages:

(A) Introduction: a new paragraph explaining the dual role of gene flow in climate adaptation: introgression of adaptive alleles vs. asymmetric introgression as a mechanism of gradual lineage replacement. This paragraph explicitly connects genome dosage differences (octoploid vs. tetraploid) to the natural directionality of backcrossing.

(B) Discussion 4.2: a new paragraph extending the discussion of asymmetric introgression into its long-term evolutionary consequences, including the potential erosion of the CN lineage's genetic distinctiveness and the risk of losing cold-adapted alleles under future climate volatility.

Introduction (new paragraph)

“Gene flow between lineages of differing ploidy can play a dual role in climate adaptation. Introgression may introduce adaptive alleles (e.g., heat tolerance loci) into a recipient lineage, facilitating its persistence under warming (Suarez-Gonzalez et al., 2018). Conversely, if introgression is asymmetric, such that one lineage's genome is disproportionately represented in admixed populations, it can drive a gradual but systematic shift in genetic composition within the contact zone—effectively functioning as a mechanism of lineage replacement without requiring complete competitive exclusion (Bartolić et al., 2024; Zohren et al., 2016). In mixed-ploidy systems, genome dosage differences create a natural directionality in backcrossing: hybrids tend to backcross more frequently with the high-ploidy parent (Bartolić et al., 2024). In the present study, we test whether such a bias exists between the octoploid FEAU and tetraploid CN lineages and examine its consequences for future distribution under climate warming.”

Discussion 4.2 (new paragraph)

“From an evolutionary standpoint, asymmetric introgression can erode the genetic distinctiveness of the minority lineage (CN) while enriching the majority lineage (FEAU) with alleles that may have been locally adapted in the CN genomic background. This could reduce the species' overall evolutionary potential, even if the FEAU lineage itself thrives—because cold-adapted alleles from the CN lineage, which may be valuable under future climate volatility (including extreme cold events), risk being diluted or lost (Exposito-Alonso et al., 2022). The directionality of introgression is also not fixed; it could shift if environmental conditions alter hybrid fitness or if the demographic balance between lineages changes. Long-term genomic monitoring of the CN–FEAU contact zone will be essential to determine whether the asymmetric gene flow documented here represents a transient phase or a persistent trajectory toward genomic homogenization.”

**Recommendations for the authors:**

**Reviewing Editor Comments:**

(RE-1) Explain how ploidy level is inferred

We invite the authors to clearly explain how the level of ploidy is being inferred (Reviewer #1).

We agree that the rationale for ploidy assignment and its relationship to allele counts needed greater clarity. This has been addressed by the comprehensive revision of Methods 2.1 described in response to R1-P1 (Part 1, Reviewer #1 Public Reviews). The revised text now presents a complete step-by-step logical chain: (i) *P. australis* has an allotetraploid base genome; (ii) all 42 markers map to a single unique chromosome in the reference genome, confirming that each marker amplifies from only one subgenome; (iii) tetraploids therefore show at most two distinguishable alleles per locus, while octoploids (autopolyploid derivatives of tetraploids) show up to four; (iv) because many samples lacked independent ploidy confirmation, we uniformly set Ploidies(mydata) <- 4 in polysat as a conservative treatment that accommodates both tetraploids (two observed copies + two null) and octoploids (four observed copies).

See the full revised text under R1-P1 (Part 1) above.

(RE-2) Common garden results are correlational

We note that the findings of the common garden experiment, although interesting, are mostly correlational (not causative) and rely on a relatively small sample size and confound lineage isolation and adaptive differentiation (both Reviewers).

We fully acknowledge this limitation. Because all octoploids belong to the FEAU lineage and all tetraploids to CN, ploidy and lineage identity are inherently confounded. This has been addressed by the four-part revision described in response to R2-P1 (Part 1, Reviewer #2 Public Reviews). Specifically:

The Abstract now frames the findings as "polyploidy-associated" rather than "rooted in polyploidy."

The Introduction now explicitly states that ploidy is correlated with—but not experimentally separable from—the broader genetic identity of each lineage.

The Discussion 4.1 title was changed to "Polyploidy-associated thermal tolerance" and the opening paragraph now presents multiple non-mutually-exclusive mechanisms rather than asserting a causal role for polyploidy.

The Discussion 4.4 now includes an expanded limitations paragraph acknowledging that ploidy was not experimentally manipulated and that common garden results should be interpreted as lineage-associated rather than ploidy-causal.

In addition, the Methods 2.3 and Discussion 4.4 revisions described in response to R1-P2 (Part 1) address the sample size concern by clarifying the experimental design and adding a dedicated acknowledgement of the limited population replication.

See the full revised text under R2-P1 and R1-P2 (Part 1) above.

**Reviewer #1 (Recommendations for the authors):**

(R1-R1) "Large morphological traits"

Line 85. Large morphological traits. Does this mean physically large? Or higher values of some trait.

We agree the original phrasing was ambiguous. We have replaced "large morphological traits" with explicit trait descriptions.

Introduction

“The octoploid FEAU lineage exhibits greater shoot height, larger leaf size, and thicker stems (K. Chen et al., 1993; Guo et al., 2025; Liu et al., 2021b, 2026; Yin et al., 2024), along with stronger salt tolerance and higher thermal tolerance”

(R1-R2) Why 2 allele copies expected for a tetraploid

Line 119. Not clear why this allele copy number is expected. A tetraploid can have up to 4 unique alleles (e.g., ABCD), not two.

This comment arises from the same conceptual gap addressed in R1-P1. The key point is that *P. australis* is an allotetraploid with two subgenomes, and our 42 markers each map to a single unique chromosome (one subgenome). Therefore, the marker only amplifies the two homologous copies from that subgenome, giving at most two distinguishable alleles. A true autotetraploid would indeed show up to four alleles—but that is not the genomic architecture of *P. australis*. The revised Methods 2.1 (see R1-P1 in Part 1) now explicitly explains this logic.

Fully addressed by the Methods 2.1 revision in R1-P1.

(R1-R3) Theoretical expectation of allele number vs. ploidy

Line 127-129. As above, this is unclear and not what we expect theoretically. If there is a reasonable number of alleles, there should be a maximum of 4 for tets, 6 for hex, and 8 for octs.

Same point as R1-R2. The reviewer's expectation (4 for tetraploids, 6 for hexaploids, 8 for octoploids) is correct for *autopolyploids* with markers that amplify all homologous copies. The discrepancy arises because *P. australis* is an allotetraploid and our markers are single-chromosome-anchored (each amplifying from only one subgenome). The revised Methods 2.1 now clarifies this distinction explicitly.

Fully addressed by the Methods 2.1 revision in R1-P1.

(R1-R4) Typo "makers"

Line 136. Should be 'markers' not 'makers'

We have performed a full-text search and corrected all instances of "makers" to "markers" in the manuscript.

Full-text search and replace.

(R1-R5) Reason for removing markers with >4 alleles

Line 142. The reason for the removal of more than 4 alleles is not clear. What about hexaploids and octoploids? They can carry 6 or 8 alleles, respectively.

We agree the original text did not adequately justify this quality-control step. In our study, the maximum expected distinguishable alleles (given the allotetraploid genome and single-chromosome-anchored markers) is two for tetraploids and four for octoploids. The observation of five or more alleles in multiple individuals is therefore diagnostic of multi-locus amplification (the marker amplifying more than one genomic locus), not of high ploidy. This is a quality-control filter, not a ploidy assignment criterion.

Methods 2.1

“During genotyping, eleven markers (including four of the five multi-mapping markers) were removed because more than ten samples exhibited more than four alleles per sample at these loci. Because the maximum number of distinguishable alleles expected under our ploidy model is two (tetraploid) to four (octoploid), the observation of five or more alleles in multiple individuals indicates that these markers amplify more than one genomic locus, rendering them unsuitable for dosage-based genotyping. This filtration is a quality-control step, not a ploidy assignment criterion.”

(R1-R6) Unclear sample sizes in common garden

Line 240. Unclear sample sizes. If these are the numbers, they are a very low level of replication expected for a common garden experiment.

Same point as R1-P2 (Public Review). Please see the full response under R1-P2 in Part 1, where we have (A) clarified the experimental design in Methods 2.3, (B) added Cohen's *d* effect sizes in Results 3.3, and (C) acknowledged the sample size limitation in Discussion 4.4.

Fully addressed by the three-part revision in R1-P2.

**Reviewer #2 (Recommendations for the authors):**

(R2-A1) De-emphasize polyploidy

De-emphasize polyploidy, as it's not manipulated in the study and is entirely confounded with the genotypes of the distinct lineages, and the putative links between polyploidy and heat tolerance are circumstantial and lacking in a clear mechanism.

We agree fully. This has been addressed comprehensively across four locations in the manuscript (Abstract, Introduction, Discussion 4.1, Discussion 4.4). See the full response under R2-P1 (Part 1) for the revised text at each location.

Fully addressed by the four-part revision in R2-P1.

(R2-A2) Emphasize the novelty of combining SDMs with experiments

Emphasize the novelty of combining SDMs with experiments (or, if I'm not up on the literature and they are more common, explain how they have been used to make new insights).

We appreciate this suggestion and agree that explicitly stating the novelty of our integrative approach strengthens the manuscript. We have added a new paragraph at the end of the Introduction.

Introduction (end, before "Here, we integrate population genomics…")

“Studies that combine species distribution models with physiological or common garden experiments remain surprisingly uncommon (but see López-Jurado et al., 2019). Such integration is essential for transforming correlative SDM projections into mechanistically grounded predictions. In the present study, we adopt this integrative approach: common garden experiments directly test growth performance under controlled conditions, heat-tolerance measurements identify the specific physiological thresholds (*T*_crit_, *T*_50_) underlying lineage-specific climate responses, and SDMs project how these experimentally documented differences translate into spatial dynamics under future warming. By linking experimental data with spatial forecasting, we move beyond correlative climate matching toward a trait-based understanding of how intraspecific variation shapes species' future distributions.”

(R2-A3) Elaborate on the importance of gene flow

Elaborate on the importance of gene flow and the potential connections between gene flow and evolving species (or lineage) geographical limits.

This has been addressed by the two new paragraphs described under R2-P3 (Part 1)—one in the Introduction on the dual role of gene flow in climate adaptation (introgression of adaptive alleles vs. asymmetric introgression as a mechanism of lineage replacement), and one in Discussion 4.2 on the long-term evolutionary consequences of asymmetric introgression.

Fully addressed by the two-part revision in R2-P3.
